# Propolis improves intestinal barrier function against *Cryptosporidium parvum* via NLRP6 inflammasome

**DOI:** 10.1128/mbio.02317-25

**Published:** 2025-10-08

**Authors:** Chang Xu, Qing He, Ziye Zhu, Kun Li

**Affiliations:** 1 College of Veterinary Medicine, Nanjing Agricultural University 70578, Nanjing, People's Republic of China; Cornell University College of Veterinary Medicine, Ithaca, New York, USA

**Keywords:** propolis, *Cryptosporidium parvum*, NLRP6, intestinal barrier, microbiota

## Abstract

Cryptosporidiosis, a significant global zoonotic disease, poses a serious health threat due to the current lack of effective and specific therapeutic agents, particularly for immunocompromised individuals and young animals. The therapeutic potential and underlying mechanisms of propolis alcohol extract (BP) and water extract (WBP) in *Cryptosporidium parvum* infection remain incompletely understood. The study assessed the cytotoxicity of BP and WBP, as well as their effects on host cell infection. For the *in vivo* phase, an immunosuppressed mouse model was employed. Fecal oocyst shedding was quantified via RT-qPCR, and therapeutic efficacy was further evaluated through intestinal histopathological examination. Serum levels of inflammatory cytokines (IL-6, IL-1β, TNF-α, IL-10) and oxidative stress markers (MDA, SOD, GSH-Px) were measured using an enzyme-linked immunosorbent assay to assess immunomodulatory and antioxidant activities. In addition, the expression of intestinal tight junction proteins (ZO-1, occludin, claudin-1) and key components of the NLRP6/caspase-1/IL-18 inflammatory pathway was analyzed by RT-qPCR and Western blot. Molecular docking analysis predicted interactions between major bioactive compounds in propolis, such as hesperidin and quercetin, and the critical adhesion protein gp40/15 of *C. parvum*. 16S rRNA sequencing revealed alterations in the intestinal microbiota, identifying key genera such as *Turicibacter* and *Ligilactobacillus*. Overall, the results of this study provide an important practical basis for the development of new anti-*C*. *parvum* drugs. Both BP and WBP significantly reduce *C. parvum* infection burden, alleviate intestinal inflammation, reduce oxidative stress, and restore intestinal barrier integrity, with BP exhibiting more pronounced antiparasitic and immunomodulatory effects.

**IMPORTANCE:**

*Cryptosporidium* is an important pathogen that causes diarrhea in infants and young children and serious diseases in patients with weakened immune function. Currently, there are no specific drugs for its treatment. This study compared the inhibitory effects of propolis extracted by different methods on *Cryptosporidium* and revealed its inhibitory mechanism. Propolis can directly target the key virulence factor gp40/15 protein on the surface of insects, interfering with the invasion and colonization of *C. parvum*. In addition, propolis enhances the anti-*C*. *parvum* immune response by activating the host’s NLRP6 inflammatome pathway and promoting the production of protective cytokines such as IL-18/IFN-γ. Studies have confirmed that propolis can simultaneously improve intestinal barrier damage and flora imbalance caused by infection. These findings provide a scientific basis for the development of propolis as a natural anti-*C*. *parvum* drug. The gp40/15 target and the NLRP6 inflammasome regulatory mechanism also offer new ideas for the research and development of anti-*C*. *parvum* drugs.

## INTRODUCTION

*Cryptosporidium*, as a significant protozoan pathogen within the phylum Apicomplexa, is an opportunistic intestinal parasite of considerable public health importance. Its primary mode of transmission is waterborne (1). Epidemiological data indicate that *Cryptosporidium parvum* infection is widespread worldwide, with human cases reported in at least 74 countries and regions to date. Molecular epidemiological studies have identified approximately 20 species and genotypes associated with human infections, among which *Cryptosporidium hominis* and *C. parvum* are the most frequently encountered in clinical settings (2). The disease predominantly affects infants and young children under the age of 5 (3, 4), although healthy adults are also susceptible to infection. While severe clinical manifestations are typically observed in individuals with compromised immune systems, epidemiological studies have also identified a notable number of asymptomatic carriers (5). A global multicenter study conducted by several international research institutions systematically analyzed pathogens responsible for moderate to severe diarrhea in children across seven regions in sub-Saharan Africa and South Asia. The findings identified *C. parvum* as one of the four leading pathogens associated with moderate to severe diarrhea in children under 2 years of age (6). Furthermore, among all diarrheal pathogens affecting children under two, *C. parvum* ranks second in terms of pathogenic prevalence, following only rotavirus (7). The life cycle of *C. parvum* involves a complex interaction between the parasite and its host (8). Following oral ingestion, infectious oocysts undergo excystation in the gastrointestinal tract under specific environmental conditions, releasing sporozoites capable of invasion (9). These sporozoites utilize apical complex-secreted adhesins to bind to receptors on the surface of intestinal epithelial cells. The invasion process is facilitated by actin cytoskeleton remodeling in the host cell (10). Once inside the host cell, the parasite activates multiple intracellular signaling pathways and establishes a parasitophorous vacuole at a specific site within the host cell membrane but outside the cytoplasmic compartment (11). This unique parasitic mechanism leads to significant alterations in the physiology of the host intestinal epithelium, primarily characterized by impaired nutrient absorption and excessive secretion (12). Cryptosporidiosis does not currently have a highly effective pharmacological treatment (13). Nitazoxanide is the only medication that has been authorized by the U.S. FDA for the treatment of this illness. However, in immunocompromised people, like AIDS patients, its therapeutic efficacy is limited (14, 15).

In recent years, traditional Chinese medicine has garnered significant attention in the field of antiparasitic drug research and development due to its multi-component and multi-target characteristics (16). Propolis, also known as bee gum, is a viscous, solid, gel-like substance formed by the mixture of plant resins collected by honey bees and secretions from their mandibular and wax glands. This substance is utilized by bees to protect against pathogenic microorganisms and to disinfect and sterilize the hive environment (17). The primary bioactive components of bee propolis are polyphenolic compounds, including phenolic acids, flavonoids, and their esters (18). Propolis has been used for centuries as a traditional natural remedy because of its antibacterial, anti-inflammatory, and metabolic-regulating qualities (19). Numerous animal and clinical studies have demonstrated that propolis exerts therapeutic effects on various diseases, such as diabetes, obesity, and non-alcoholic fatty liver disease (20, 21). Moreover, some studies have indicated that propolis can effectively eliminate surface parasites in cattle and sheep and also resist trypanosoma (22).

A dynamic and intricate ecosystem is made up of the intestinal microbiota. Symbiotic microorganisms residing in the gastrointestinal tract can influence the susceptibility to parasitic infections. As a critical component of the intestinal barrier, alterations in the composition of the intestinal microbiota may enhance resistance to parasitic infections in the intestine and other mucosal tissues by reducing parasite virulence or adhesion (23). Metabolic products generated by the intestinal microbiota may directly inhibit parasite growth and virulence or indirectly enhance the host’s innate immune response by improving epithelial barrier integrity and modulating the production of pro-inflammatory and anti-inflammatory cytokines (24, 25). A balanced intestinal microbiota is essential for maintaining immune homeostasis in the host. However, parasitic infections often lead to characteristic dysbiosis, marked by an increased abundance of Proteobacteria and a decreased abundance of Bacteroidetes (26, 27). This ecological imbalance, often characterized by the overgrowth of the Enterobacteriaceae family, is frequently accompanied by impaired function of Paneth cells, further exacerbating disease progression (28).

Therefore, this study aimed to investigate the inhibitory effects of propolis, a traditional Chinese medicine, on *C. parvum*. By evaluating the expression levels of intestinal barrier proteins and immune-related proteins, and using molecular docking to study the inhibitory effect of propolis on *C. parvum* adhesion protein gp40/15, combined with intestinal flora analysis, this study provides the first evidence demonstrating the inhibitory effect of propolis on *C. parvum* infection.

## RESULTS

### Quantitative immunofluorescence analysis of *C. parvum* within cells

**Fig 1:**
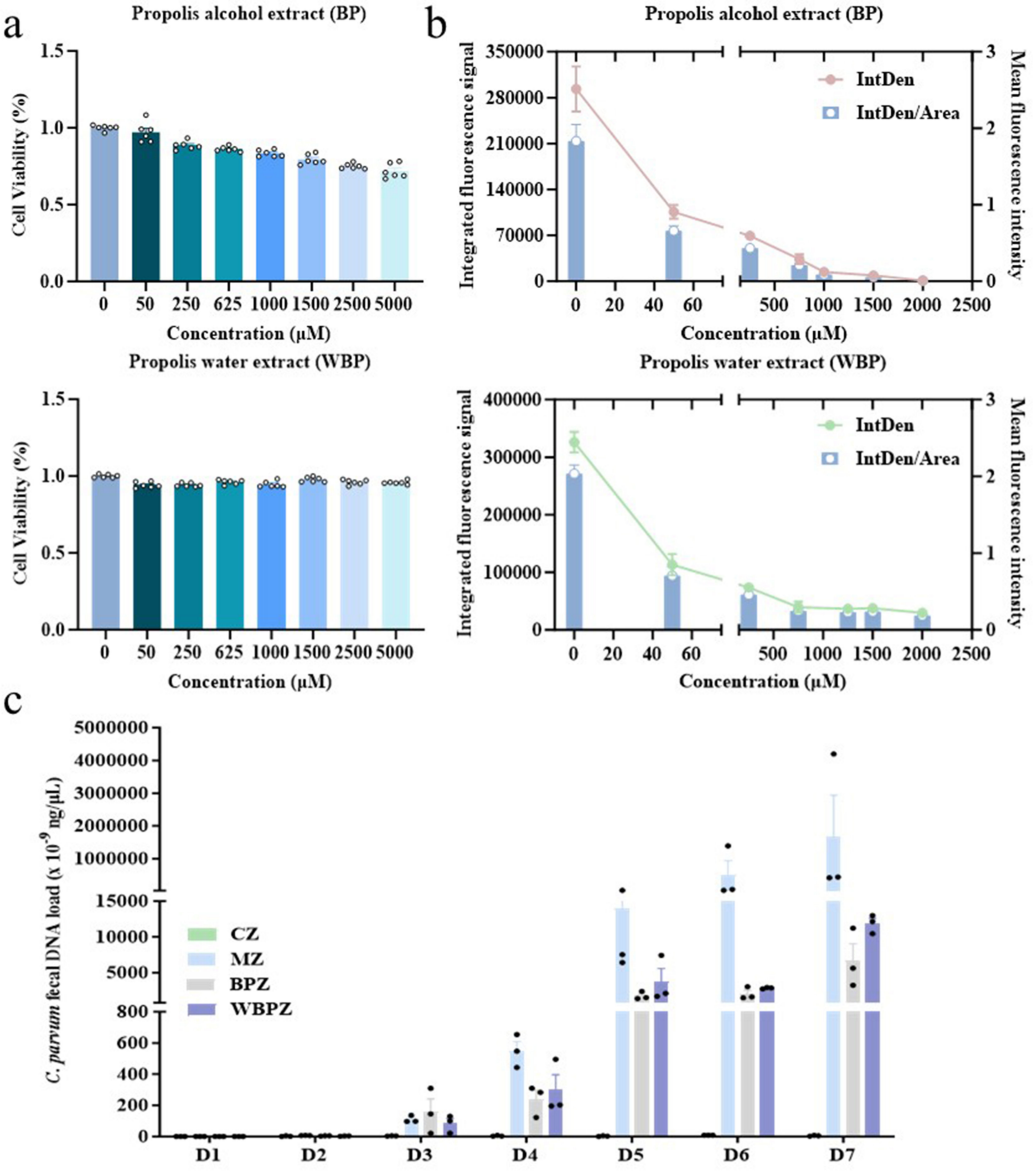
Propolis inhibits the growth of *Cryptosporidium parvum in vivo* and *in vitro*. (**a**) Cytotoxicity study of propolis alcohol extract and water extract; (**b**) *Cryptosporidium* immunofluorescence analysis; (**c**) quantitative results of mouse feces. Statistical significance is indicated as **P < 0.01, ***P < 0.001, ****P < 0.0001. Data are presented as mean ± SEM.

The results of the cytotoxicity assay demonstrated that BP exhibited a pronounced dose-dependent cytotoxic effect within the concentration range of 0–5,000 µM. As the concentration increased across the gradient (0, 50, 250, 625, 1,000, 1,500, 2,500, and 5,000 µM), the viability of the cells gradually decreased. Notably, even at the highest tested concentration of 5,000 µM, the cell survival rate remained approximately 80%. In contrast, WBP did not exhibit significant cytotoxicity within the same concentration range. The cell viability across all WBP-treated groups remained consistently around 100%, with no observable toxic effects (Fig. 1a). Based on these cytotoxicity findings, to ensure experimental safety, a maximum treatment concentration of 2,000 µM was selected for subsequent immunofluorescence experiments.

Immunofluorescence data indicated that both BP and WBP exhibited inhibitory effects at concentrations as low as 50 µM. With increasing drug concentrations, both compounds demonstrated enhanced anti-*C*. *parvum* activity in a concentration-dependent manner. Particularly, the BP group showed nearly complete inhibition at 2,000 µM, with fluorescence intensity reduced by approximately 300-fold compared to the control group. In comparison, WBP reached a plateau in its inhibitory effect within the concentration range of 750–2,000 µM, maintaining an inhibition level comparable to that of 750 µM BP (Fig. 1b).

### Quantitative analysis of *C. parvum* in the feces of mice

The *C. parvum* 18S rRNA gene in the feces of four groups (CZ, MZ, BPZ, and WBPZ, 100 mg/kg, which was confirmed as a safe dose in the previous toxicity test) of mice was quantitatively analyzed using RT-qPCR. The results indicated that no *C. parvum*-specific nucleic acid was detected in the normal control group (CZ) throughout the entire experimental period. In the model group (MZ), parasite excretion began on the third day post-infection, and the parasite load increased rapidly, peaking at 1.68 × 10³ ng/μL on the seventh day. Notably, both treatment groups (BPZ and WBPZ) exhibited significant anti-*C*. *parvum* efficacy. Starting from the fifth day of treatment, the parasite load in these two groups was markedly lower than that in the model group. By the end of the experiment, the *C. parvum* load in the model group remained relatively high, whereas the loads in the BPZ group (6.7 × 10^−^⁶ ng/μL) and the WBPZ group (1.19 × 10^−^⁵ ng/μL) were significantly reduced compared to the model group (*P* < 0.001). However, the number of fecal oocysts in the propolis water extract treatment group (WBPZ) was twice that of the alcohol extract group (BPZ). Particularly, the final parasite load in the model group was approximately 200 times higher than the average of the two treatment groups (Fig. 1c).

### Serum-related index detection

To systematically evaluate the regulatory effects of propolis alcohol extract (BP) and water extract (WBP) on hosts infected with *C. parvum*, changes in inflammatory factors and antioxidant indicators in serum were measured using the enzyme-linked immunosorbent assay (ELISA) method. Experimental results indicate that *C. parvum* infection significantly activates pro-inflammatory responses, as evidenced by markedly elevated serum levels of IL-6, IL-1β, and TNF-α in the model group compared to the normal control group. Following treatment with BPZ and WBPZ, these pro-inflammatory cytokines exhibited a reduction to varying extents.

**Fig 2:**
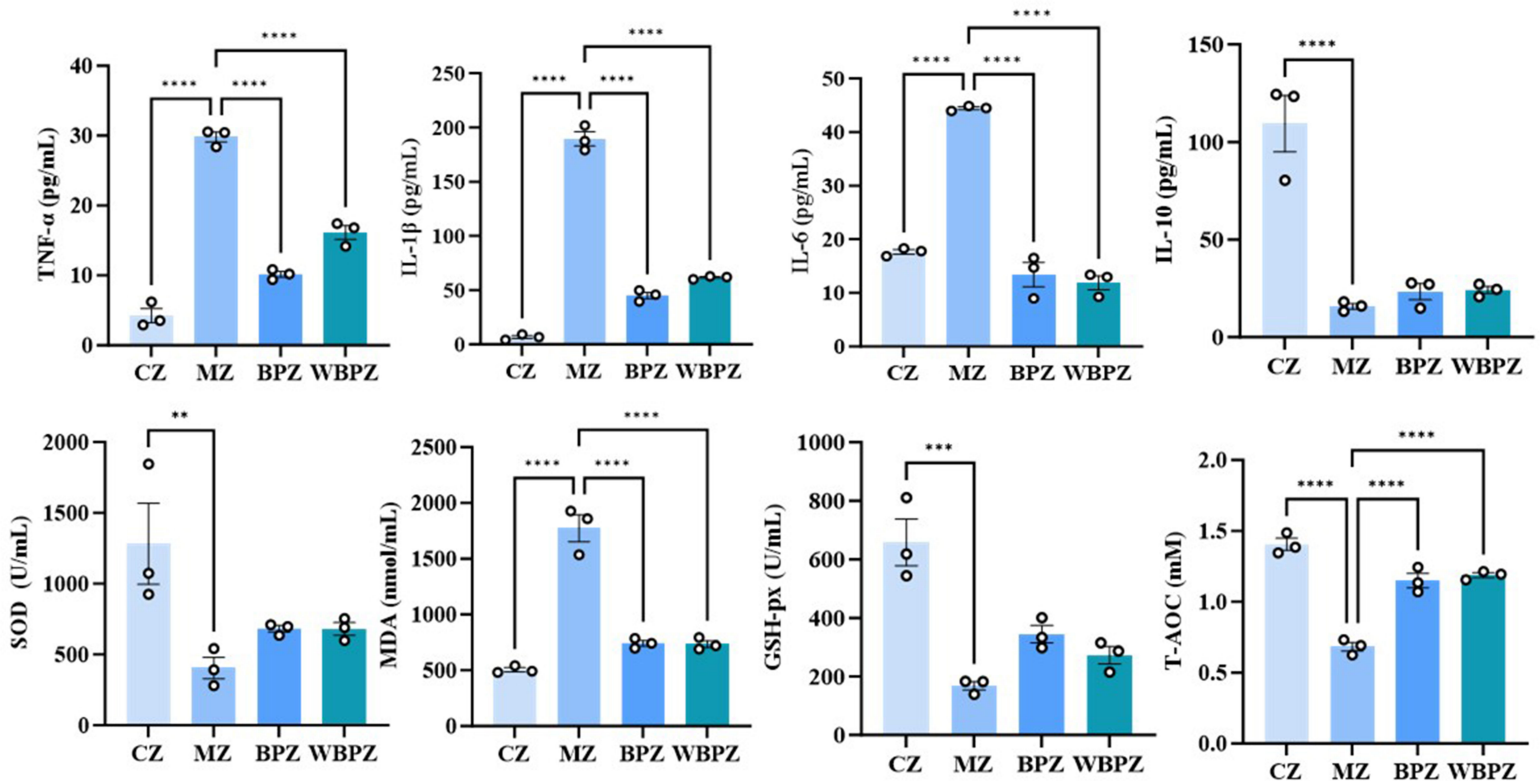
Measurement of inflammatory factors and oxidative stress indicators in mouse serum. Inflammatory factors include IL-10, IL-6, IL-1β, and TNF-α. Oxidative stress indicators include MDA, SOD, GSH-px, and T-AOC. Statistical significance is indicated as **P < 0.01, ***P < 0.001, ****P < 0.0001. Data are presented as mean ± SEM.

Regarding oxidative stress markers, *C. parvum* infection was found to impair the host’s antioxidant capacity. In particular, the model group exhibited significantly lower levels of glutathione peroxidase (GSH-Px), superoxide dismutase (SOD), and total antioxidant capacity (T-AOC) than the control group, while the level of malondialdehyde (MDA), a marker of lipid peroxidation, was significantly elevated. Administration of BPZ and WBPZ partially restored antioxidant capacity, with both treatment groups showing significantly reduced MDA levels, approaching those observed in the control group (Fig. 2).

### Analysis of intestinal pathological sections

**Fig 3:**
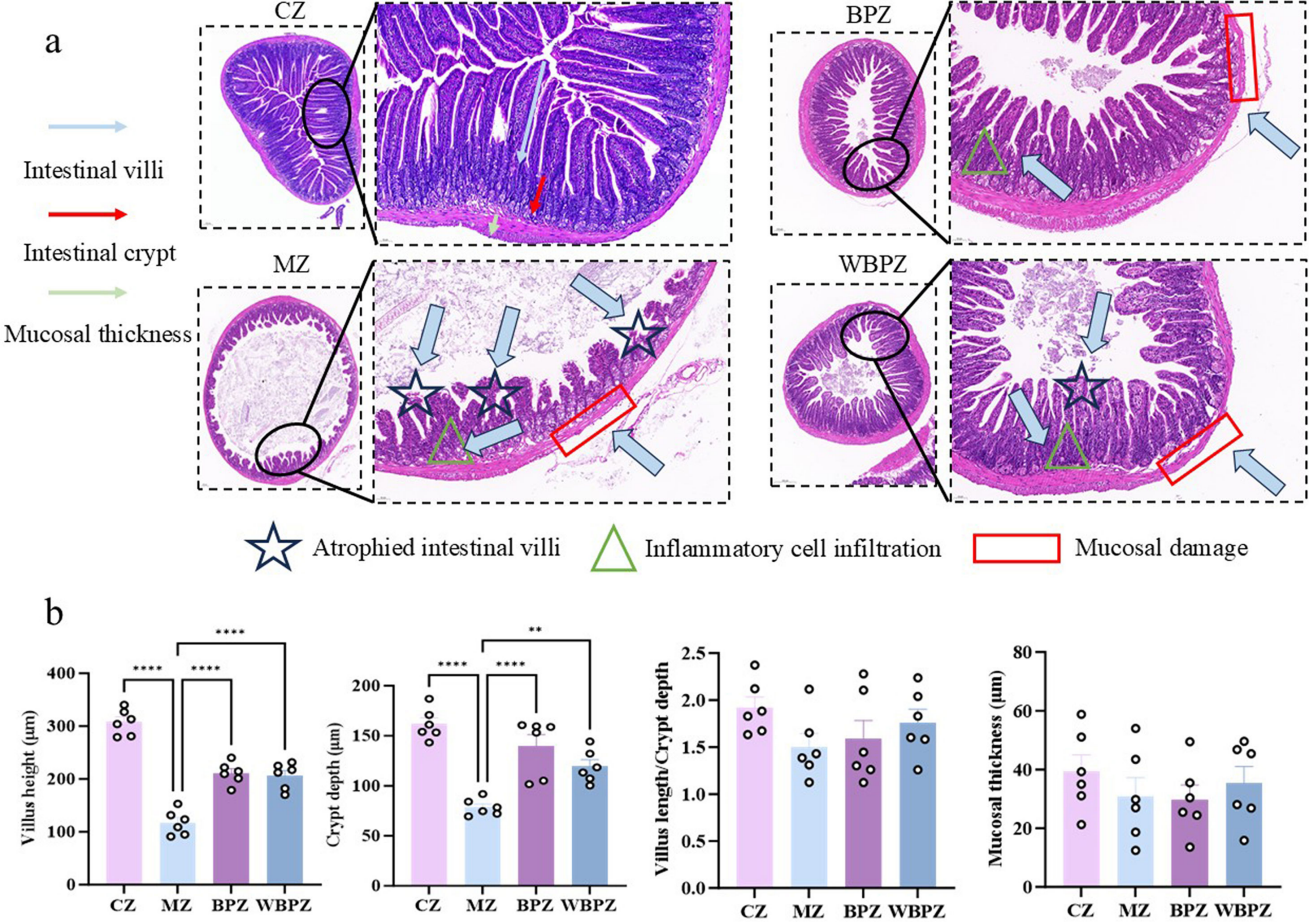
Analysis of intestinal pathological sections in mice. (**a**) HE staining section of mouse jejunum; (**b**) height of mouse intestinal villi, depth of intestinal crypts, intestinal villi/intestinal crypts, and thickness of intestinal mucosa. Statistical significance is indicated as **P < 0.01, ***P < 0.001, ****P < 0.0001. Data are presented as mean ± SEM.

The histopathological analysis of intestinal tissues following HE staining revealed distinct morphological differences among the experimental groups. In the normal control group (CZ), the intestinal mucosa exhibited an intact structure, with well-aligned and elongated villi, and no evidence of inflammatory cell infiltration in the lamina propria. In contrast, the *C. parvum* infection model group (MZ) displayed typical signs of intestinal pathology, including marked villous atrophy, reduced villus height, and localized fusion of villi. Additionally, inflammatory cell infiltration was evident within the mucosal layer, accompanied by disarrayed epithelial cell arrangement and focal mucosal defects. In the treatment group administered with water extract of propolis (WBPZ), there was a noticeable improvement in mucosal injury, with partial restoration of villus height, decreased inflammatory infiltration, and largely preserved mucosal integrity (Fig. 3a ). Notably, the group treated with alcohol extract of propolis (BPZ) demonstrated the most pronounced protective effect (Fig. 3b). The villus morphology in this group closely resembled that of the control group, with minimal inflammatory cell infiltration, orderly arranged mucosal epithelial cells, and no apparent structural defects.

### Analysis of intestinal barrier and inflammation-related proteins

**Fig 4:**
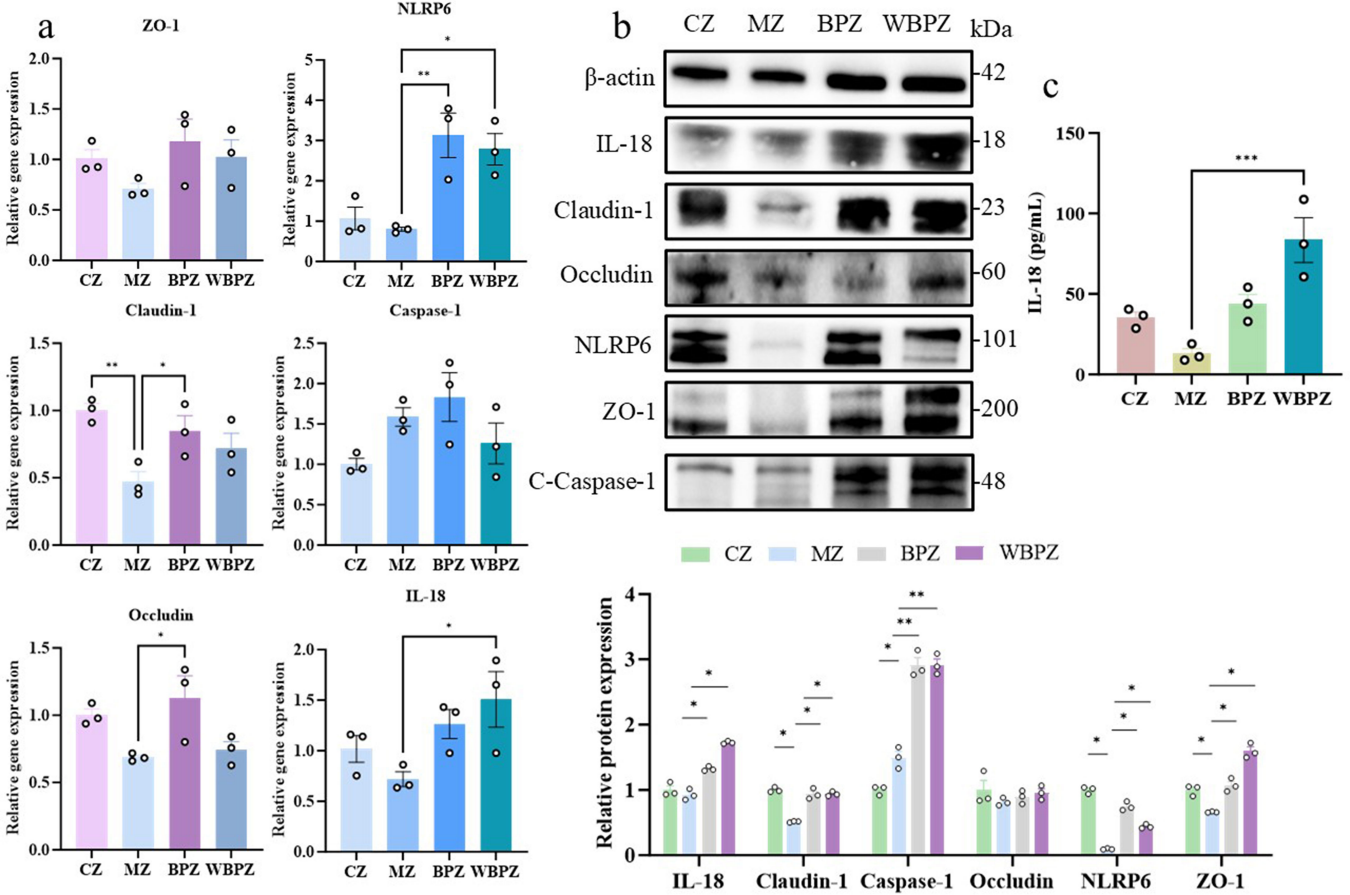
Determination of gene and protein contents related to intestinal barrier and inflammation in mice. (**a**) RT-qPCR was used to analyze the expression levels of intestinal barrier and inflammatory genes. (**b**) Western blot was used to analyze the expression levels of intestinal barrier and inflammatory proteins. (**c**) ELISA was used to determine the expression level of IL-18 in mouse serum. Statistical significance is indicated as **P < 0.01, ***P < 0.001, ****P < 0.0001. Data are presented as mean ± SEM.

We used Western blot analysis and qPCR to assess the impact of *C. parvum* infection on the intestinal inflammasome pathway and epithelial barrier function in mice. According to the findings, the infected model group (MZ) had immune dysregulation, as evidenced by significantly lower levels of NLRP6 and IL-18 expression than the normal control group (CZ), as well as less caspase-1 activation. These findings suggest that *C. parvum* infection may modulate the host immune response through regulation of the inflammasome pathway. The MZ group exhibited a significant downregulation in tight junction-associated protein expression, such as claudin-1, occludin, and ZO-1, which suggests that the infection weakens the intestinal epithelial barrier’s structural integrity. Notably, following treatment with propolis alcohol extract (BPZ) and water extract (WBPZ), the expression levels of these proteins demonstrated varying degrees of recovery. Inflammasome-related molecule expression was restored, and tight junction protein levels were significantly elevated (Fig. 4).

### The structural composition of intestinal microorganisms

**Fig 5:**
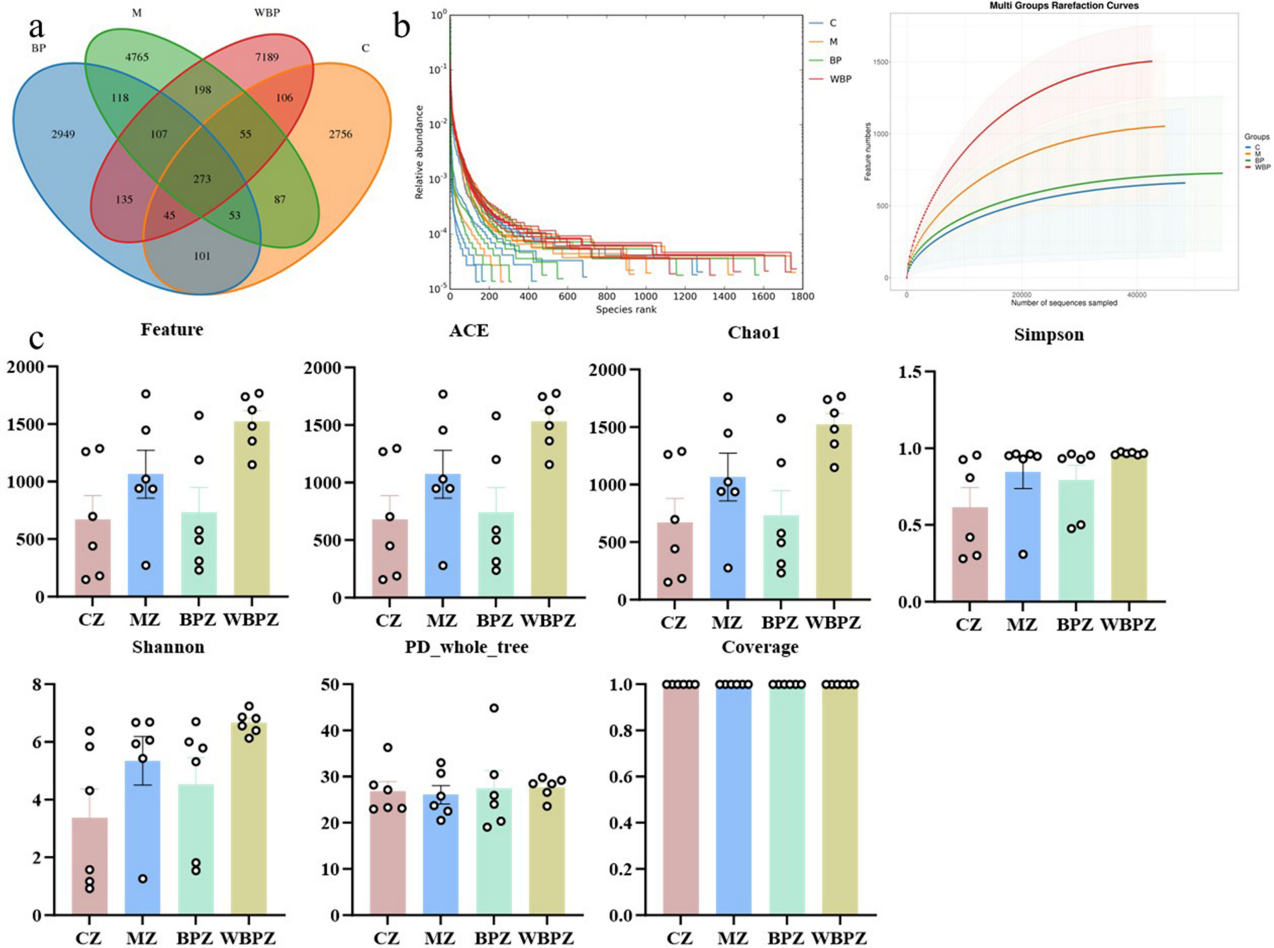
Sequencing results of the mouse intestine. (**a**) OTU composition Venn graph; (**b**) sparse curve and abundance level curve; (**c**) α-diversity analysis, including feature, ACE, Chao1, Simpson, Shannon, PD_whole_tree, and coverage. Statistical significance is indicated as **P < 0.01, ***P < 0.001, ****P < 0.0001. Data are presented as mean ± SEM.

The original sequences obtained for each sample ranged from 79,000 to 81,000. Following quality control, denoising, sequence splicing, and removal of chimeric sequences, the number of usable sequences varied between 42,000 and 74,000 across samples. Cluster analysis was performed on all samples, resulting in a total of 18,664 operational taxonomic units (OTUs) identified across the four experimental groups: 5,658 OTUs in the model group, 3,476 in the control group, 8,108 in the WBPZ group, and 3,781 in the BP group. Among these, 273 OTUs were shared across all four groups (Fig. 5a). The sparse curve and abundance grade curve indicate that the sequencing depth is sufficient (Fig. 5b). The α-diversity index revealed no significant differences in microbial richness or evenness within the groups (Fig. 5c).

**Fig 6:**
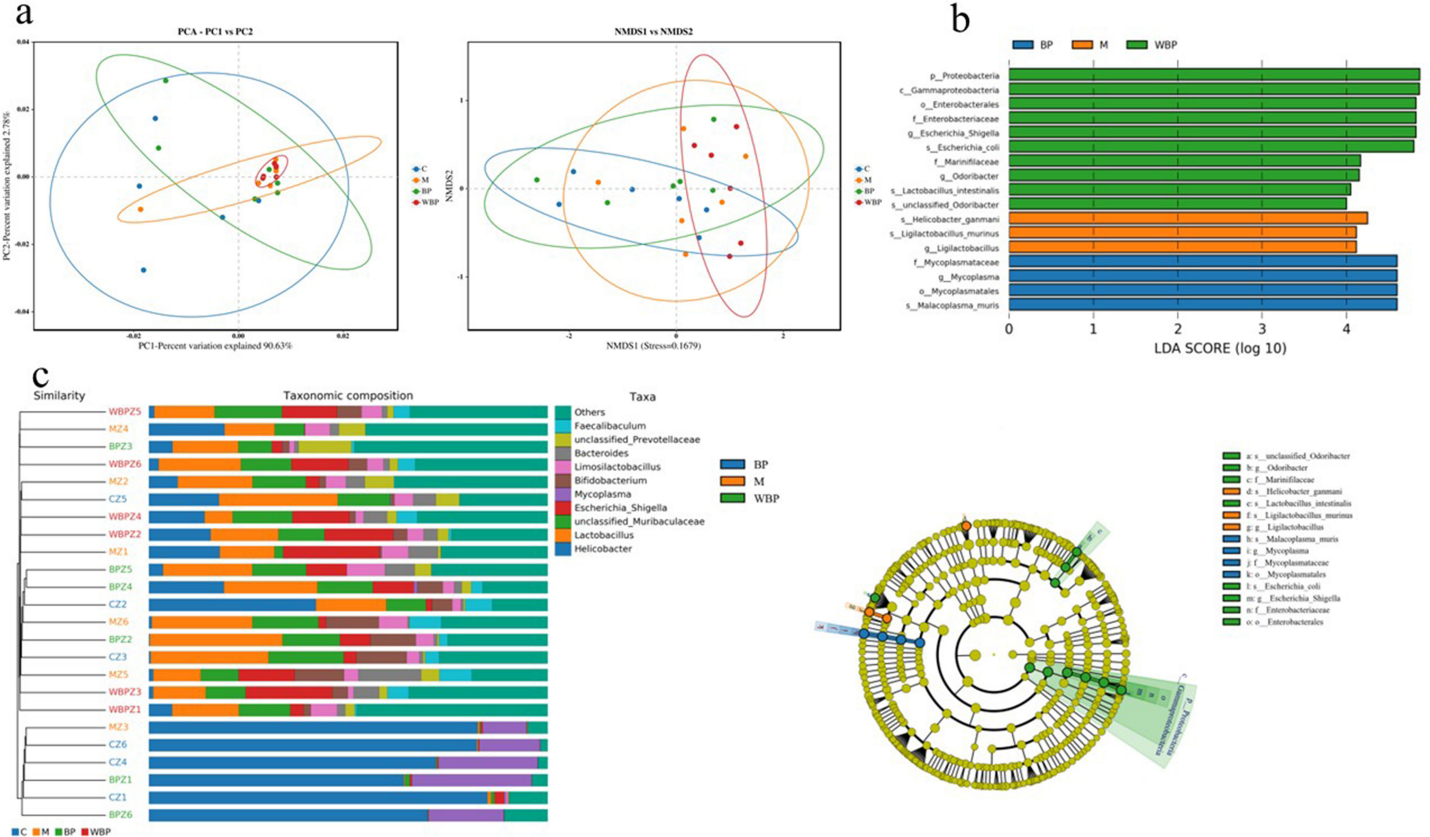
Microbiota structure analysis of mice in different groups. (**a**) β-Diversity analysis, including PCA and NMDS; (**b**) LEfSe analysis of samples between groups, including LEfSe analysis of evolutionary branching and LDA value distribution; (**c**) UPGMA cluster tree combined with bar chart.

**Fig 7:**
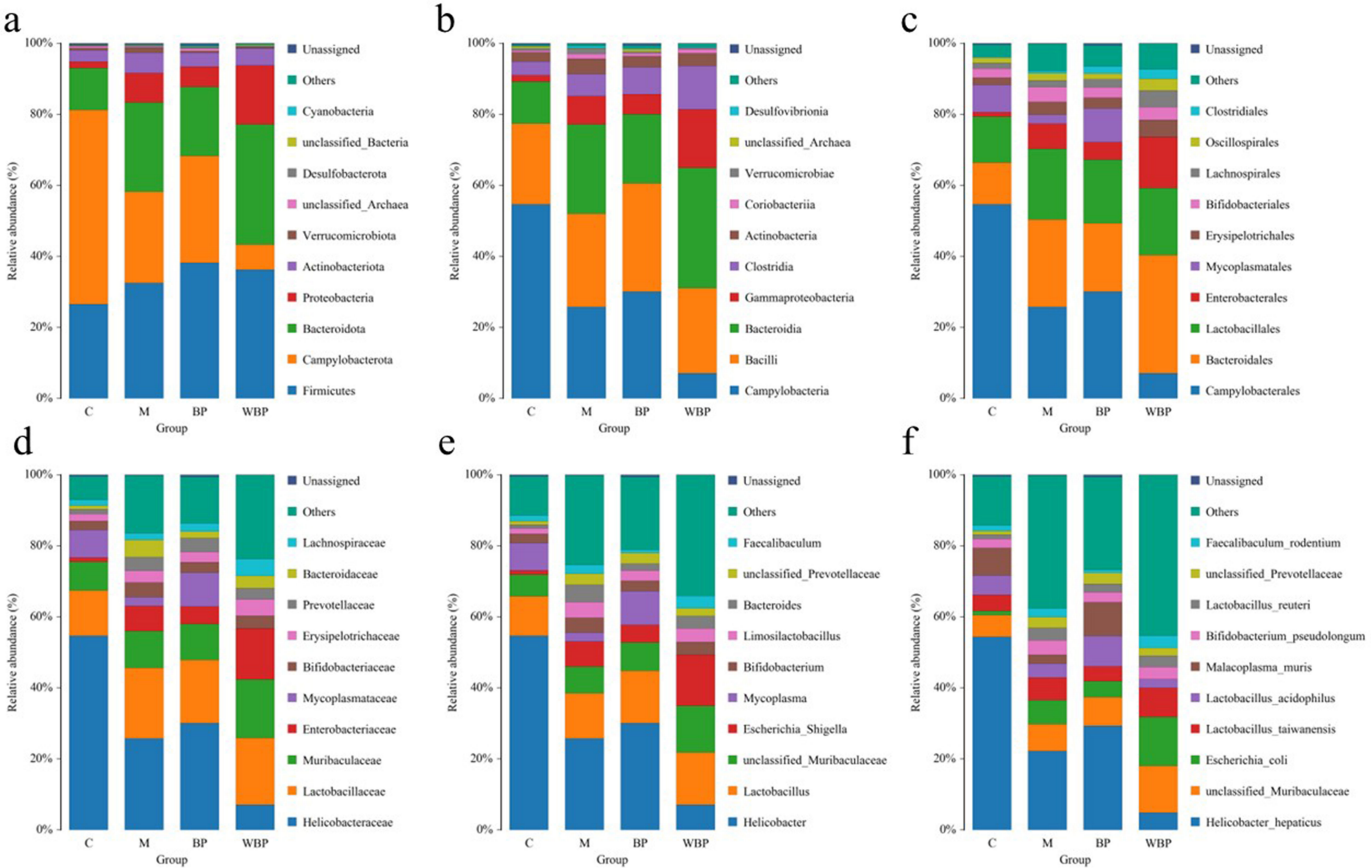
A diagram of the structural composition of the intestinal flora in mice at different levels. (**a**) Phylum, (**b**) class, (**c**) order, (**d**) family, (**e**) genus, and (**f**) species.

To evaluate the overall variations in microbial community structure among different treatment groups, beta-diversity analysis was carried out using principal component analysis (PCA), non-metric multidimensional scaling (NMDS) (Fig. 6a), and unweighted pair group method with arithmetic mean (UPGMA) (5c). LEfSe analysis identified 17 distinct bacterial taxa that exhibited significant variation across six taxonomic levels (Fig. 6b). At the phylum level, the dominant bacterial taxa varied across groups. Except for the WBPZ group, the top three phyla in the remaining three groups (control, model, and BPZ) were Campylobacterota (54.64%, 25.76%, and 30.06%, respectively), Firmicutes (26.54%, 32.47%, and 38.18%, respectively), and Bacteroidota (11.81%, 25.08%, and 19.47%, respectively). In the WBPZ group, the dominant phyla were Firmicutes (36.28%), Bacteroidota (33.84%), and Proteobacteria (16.57%). Additionally, Actinobacteriota accounted for 3.15% in the control group, while Proteobacteria represented 5.7% and 8.25% in the model and BPZ groups, respectively. In the BPZ group, Actinobacteriota (3.87%) and Proteobacteria (5.72%) were also present at notable proportions. The WBPZ group contained 7.07% Campylobacterota and 4.07% Actinobacteriota. Further analysis at the genus level revealed that *Lactobacillus* accounted for more than 10% in all four groups. *Unclassified_Muribaculaceae* represented 13.16% of the microbial composition in the WBPZ group and approximately 7% in each of the other three groups (Fig. 7).

### Intestinal differential microbiota

**Fig 8:**
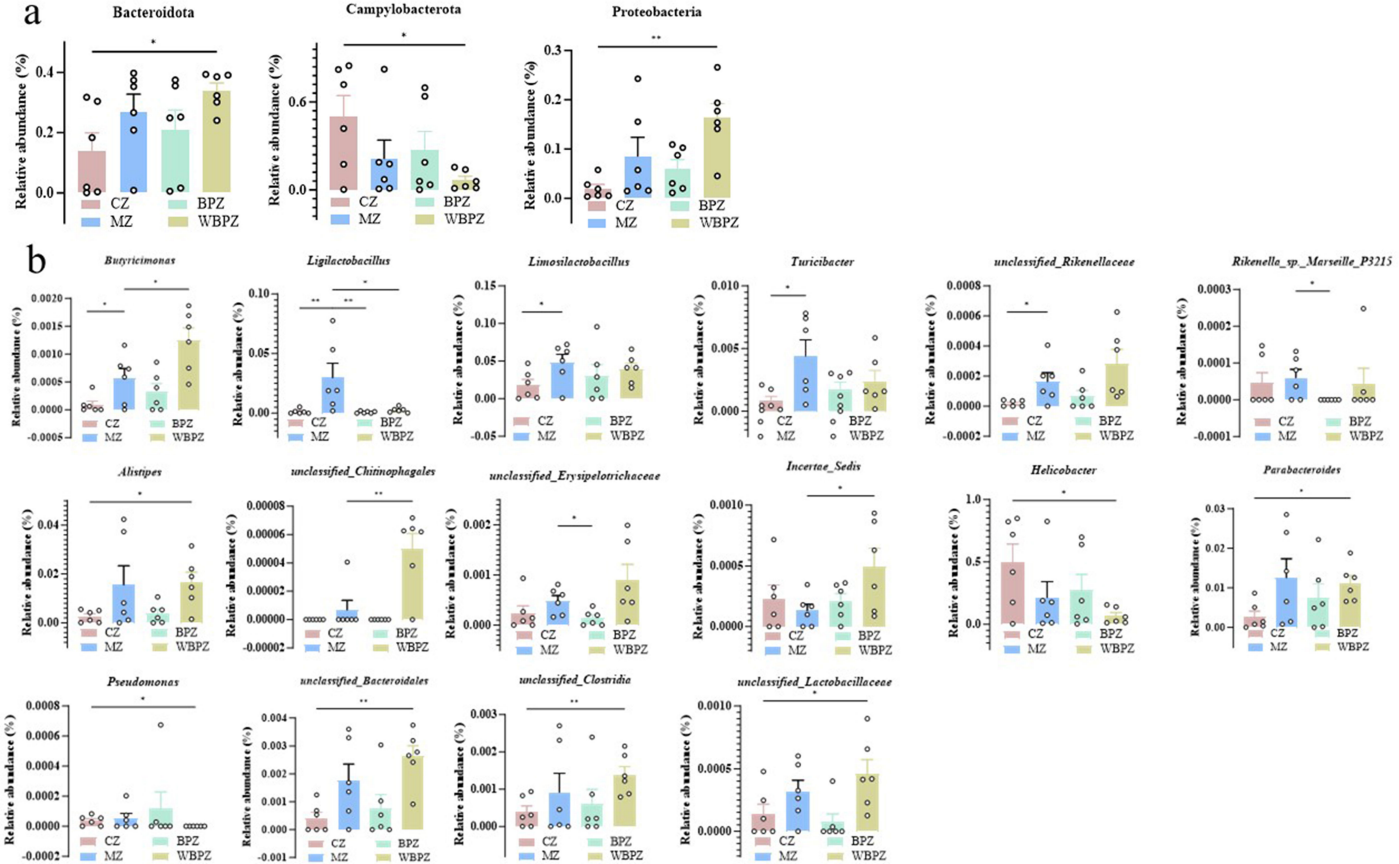
Analysis of differences in intestinal microbiota in mice. (**a**) Phylum and (**b**) genus. Statistical significance is indicated as **P < 0.01, ***P < 0.001, ****P < 0.0001. Data are presented as mean ± SEM.

The relative abundance of all bacterial taxa obtained from sequencing was reanalyzed, revealing a total of 3 phyla and 16 genera exhibiting statistically significant differences (Fig. 8). Among these, the relative abundances of several genera, including *Butyricimonas, Ligilactobacillus, Limosilactobacillus, Turicibacter, unclassified_Rikenellaceae, Rikenella_sp._Marseille_P3215, Alistipes, unclassified_Erysipelotrichaceae, Parabacteroides, unclassified_Clostridia,* and *unclassified_Lactobacillaceae*, were markedly higher in the model group compared to the control (CZ) and treatment (MZ) groups. Conversely, the abundances of *Incertae_Sedis* and *Helicobacter* were notably reduced in the model group. Notably, the microbial alterations observed in the WBPZ group showed a degree of similarity to those in the MZ group.

### Metabolic pathways of intestinal flora

**Fig 9:**
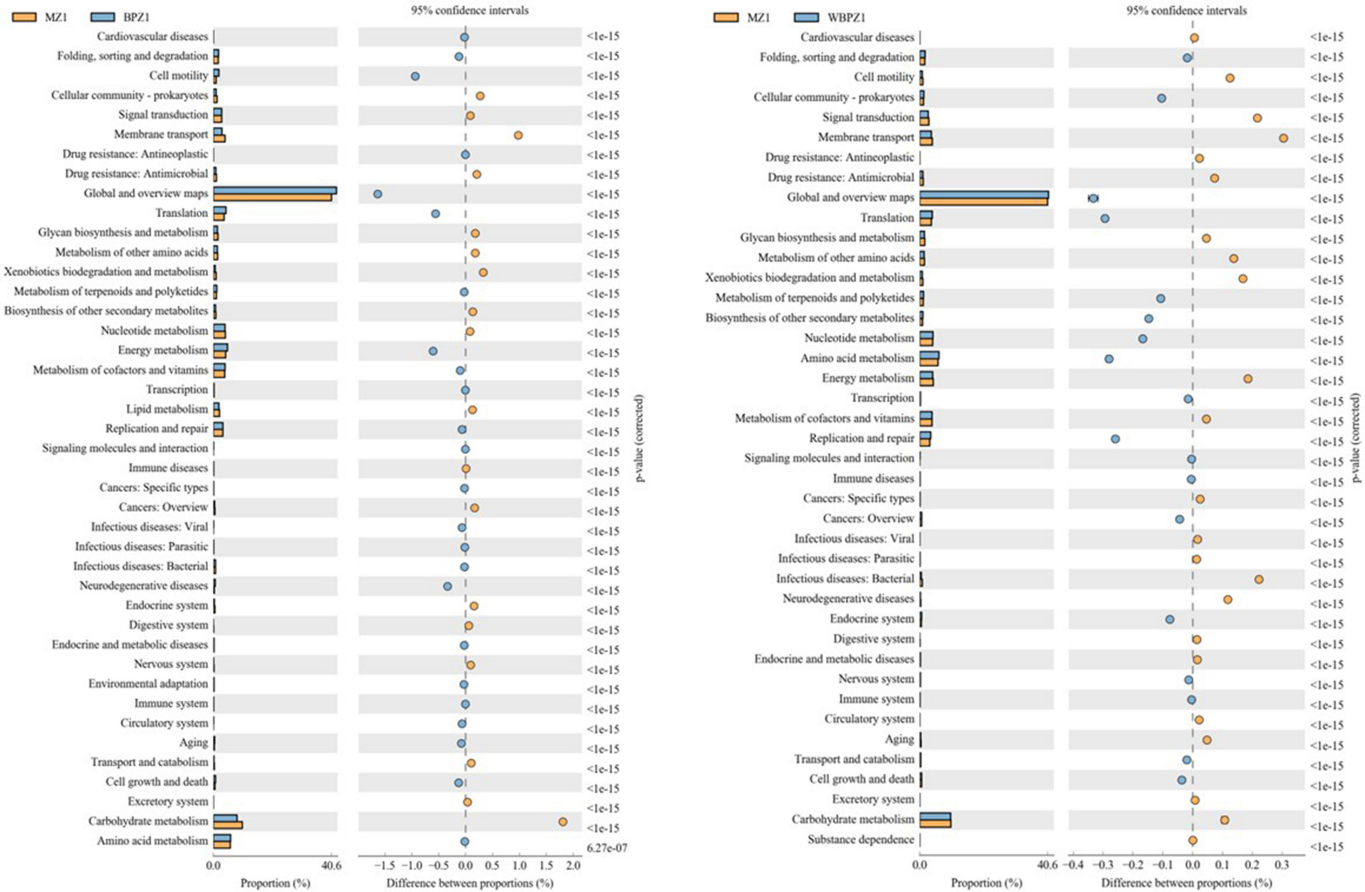
Functional analysis of nice microbiota in different groups.

Compared with the BPZ group, the activity of the microbiota in membrane transport and carbohydrate metabolism-related pathways in the MZ group was significantly enhanced, while the BPZ group enriched functional pathways such as cell motility and global metabolic mapping. It is worth noting that when compared with the WBPZ group, the microbiota in the MZ group showed excessive activation of signal transduction, membrane transport, energy metabolism, and bacterial infection-related pathways. In contrast, the microbial community of the WBPZ group is more inclined to participate in basic metabolic activities such as global metabolic regulation, translation processes, amino acid metabolism, and replication and repair of genetic material (Fig. 9).

### The components of propolis are bonded with adhesive proteins gp40/15

**Fig 10:**
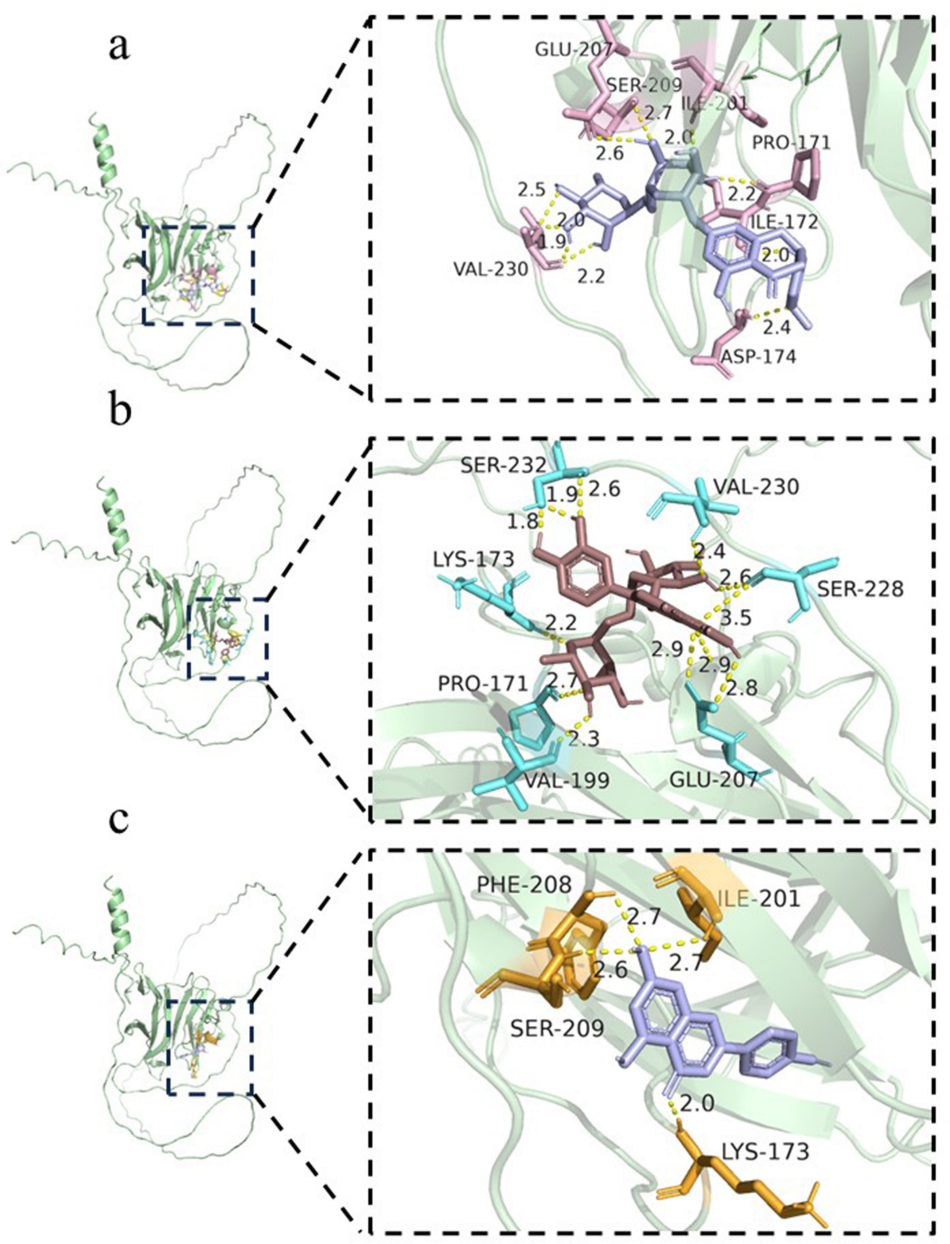
The flavonoids of propolis are conjugated with the *Cryptosporidium* adhesion protein gp40/15 molecules. (**a**) Hesperidin, (**b**) rutin, and (**c**) apigenin.

The molecular docking results demonstrated that the binding energies of the nine compounds with gp40/15 were all below −5.0 kcal/mol (Table 1). Among these, three compounds exhibited relatively lower binding energies and formed stable hydrogen bond interactions with the protein. Hesperidin displayed a binding energy of −9.6 kcal/mol with gp40/15 and formed hydrogen bonds with seven amino acid residues, including GLU-207, with bond distances ranging from 1.9 to 2.7 Å (Fig.10a). Rutin exhibited a binding energy of −7.7 kcal/mol and formed hydrogen bonds with seven amino acid residues, such as SER-232, with distances ranging from 1.8 to 3.5 Å (Fig. 10b). Apigenin showed a minimum binding energy of −7.6 kcal/mol and formed hydrogen bonds with four residues, including PHE-208, at a distance of 2.0 Å. SP-174 formed hydrogen bonds with the hydroxyl groups of the side chains of THR-228 at distances ranging from 2.0 to 2.7 Å (Fig. 10c).

**TABLE 1:** Molecular docking analysis of flavonoids in propolis with *C. parvum* GP40/15 adhesion protein

Foxtail flavonoid	Binding energy (kcal/mol)
Acacetin	−7.6 (no force)
Apigenin	−7.6
Cinnamic acid	−5.1
Ferulic acid	−5.4
Hesperidin	−9.6
Luteolin	−7.4
Quercetin	−7.4
Resveratrol	−6.7
Rutin	−7.7

## DISCUSSION

Our research demonstrated that both the alcohol and water extracts of propolis exhibited notable antiparasitic activity in the *C. parvum* infection model, significantly reducing parasite load in both cultured cells and mouse feces. This effect may be attributed to the high content of phenolic compounds (e.g., flavonoids and phenolic acids) present in the extracts (18). The alcohol extract showed superior antiparasitic efficacy, potentially due to the presence of lipophilic constituents such as terpenoids and aromatic acids, which can more readily penetrate cell membranes and exert stronger intracellular effects (29). In contrast, the polar components in the aqueous extract, such as polysaccharides, may contribute to the reduction of fecal parasite excretion through mechanisms such as intestinal immune modulation or competitive inhibition of parasite adhesion. The reduction in parasite burden was also reflected in histopathological changes observed in intestinal tissue sections. Propolis treatment significantly alleviated villus atrophy induced by infection. Specifically, the average villus height in the alcohol extract-treated group (BPZ) recovered to 211 ± 20.6 µm, while that in the water extract-treated group (WBPZ) reached 206 ± 24.5 µm, compared to 117.1 ± 23.53 µm in the model group. These findings indicate a restoration of the intestinal absorptive surface area and a marked improvement in nutrient absorption capacity. Functionally, this recovery was closely associated with increased activity of brush border enzymes, which enhances the active transport of nutrients such as glucose (30). These results collectively indicate that *C. parvum* infection has caused significant damage to the integrity of the intestinal barrier.

We further analyzed the level of systemic inflammatory response of the host after infection through serological analysis. Elevated levels of GSH-Px and SOD activity were found in serum antioxidant profiles, while MDA, a sign of oxidative stress, significantly decreased (31). The total antioxidant capacity approached that of the control group. Analysis of inflammatory cytokines indicated that as parasite clearance improved, serum levels of pro-inflammatory cytokines IL-1β, TNF-α, and IL-6 decreased, while the anti-inflammatory cytokine IL-10 increased.

The results of RT-qPCR and Western blotting (WB) demonstrated that *C. parvum* infection impaired intestinal barrier-related proteins, whereas propolis treatment restored the integrity of intestinal tight junctions. Further investigation into the relationship between *C. parvum* infection and the integrity and inflammatory response of the host intestinal barrier revealed that *C. parvum* interacts with host intestinal epithelial cells via its surface gp40/gp15 adhesins (32), resulting in the disruption of tight junction proteins, including ZO-1, occludin, and claudin-1. This degradation is primarily mediated by cysteine proteases secreted by the parasite, such as CP30, which specifically cleave the C-terminal PDZ-binding domain of ZO-1 (33). Molecular docking results indicated that various flavonoids present in propolis exhibit strong binding affinity toward the *C. parvum* adhesion protein GP40/15. These bioactive compounds may effectively reduce the adhesion capacity of the pathogen to host cells by competitively binding to key active sites on the GP40/15 protein and interfering with its interaction with intestinal epithelial cell receptors. This mechanism not only diminishes the invasiveness of *C. parvum* but may also alleviate intestinal mucosal injury and the disruption of intestinal tight junctions by blocking the initial stages of infection.

In typical parasitic infection models, activation of the NLRP6 inflammasome pathway is commonly observed, characterized by upregulated expression of NLRP6, caspase-1, and IL-18 (31, 34). However, *C. parvum* infection exhibits a distinct immunosuppressive profile, but similar phenomena have also occurred in other parasitic infections (35). Our findings revealed that *C. parvum* infection significantly downregulated NLRP6 expression, impeded caspase-1 activation, and consequently inhibited the processing and secretion of IL-18. This immune evasion mechanism facilitates parasite colonization within the host intestine. The activation of caspase-1 by NLRP6 occurs through the assembly of inflammasomes, which subsequently cleave pro-IL-18 to generate mature IL-18 (36). As a critical regulator of Th1-type immune responses, IL-18 synergizes with IL-12 to enhance IFN-γ production by NK cells, CD4+ Th1 cells, and CD8+ T cells (37). During *C. parvum* infection, IFN-γ contributes to host defense by activating macrophage and epithelial cell defense mechanisms, inhibiting parasite proliferation, and facilitating pathogen clearance. *C. parvum* may evade host immunity by disrupting inflammasome assembly. Treatment with propolis has been shown to enhance NLRP6 inflammasome activation, thereby aiding in the control of infection.

The intestinal epithelium is essential to host defense because it acts as the main immunological and physical barrier against pathogenic invasion (38). Intestinal parasites interact with the microbial community in this complex environment, affecting the balance between the gut microbiota and the host. Through its own activity and metabolic products, the gut microbiota can have an impact on parasite survival. Intestinal parasites, on the other hand, constantly release and excrete different chemicals that can alter the intestinal environment and affect the gut microbiota’s makeup (23).

Our study revealed that the abundance of Bacteroidota increased in both the model group and the propolis water extract treatment group, whereas the abundance in the propolis alcohol extract treatment group was similar to that in the control group. Certain members of this phylum are capable of degrading complex polysaccharides to produce short-chain fatty acids (SCFAs) (39), which exert beneficial effects, including anti-inflammatory activity and the maintenance of intestinal barrier integrity (40). However, some species within the Bacteroidota phylum may act as opportunistic pathogens, and their excessive proliferation may be associated with dysbiosis of the intestinal microecosystem (41). In the *C. parvum* infection model group (MZ), the abundance of *Turicibacter* was significantly elevated. As a genus closely associated with intestinal inflammatory conditions (42), the increased presence of *Turicibacter* may serve as a potential indicator of *C. parvum* infection. Furthermore, the model group had a higher abundance of *Ligilactobacillus*. Probiotics are essential for preserving intestinal homeostasis and enhancing host health (43), but an overabundance of them can have negative consequences. The gut microbiome constitutes a highly complex ecosystem whose stability relies on the dynamic balance among various microbial populations. When *Ligilactobacillus* exhibits abnormal proliferation in the model group, it intensifies competition among microbial species, thereby inhibiting the growth of other symbiotic bacteria and reducing microbial diversity. Excessive probiotic activity may result in overproduction of SCFAs through enhanced fermentation, leading to a significant decrease in intestinal pH, which may compromise the integrity of the epithelial barrier (44). Moreover, excessive stimulation by probiotics may hyperactivate the immune system, particularly under conditions of infection, potentially exacerbating the inflammatory response (45). The observed increase in *Ligilactobacillus* abundance in the model group may represent an abnormal compensatory mechanism by the host in response to *C. parvum* infection. Although short-term overgrowth of probiotics may aid in pathogen resistance, it may disrupt the intestinal microecological balance over time. The regulatory effects observed in the propolis treatment groups suggest that propolis may help restore and maintain the balance of the intestinal microbiota.

### Conclusion

This study systematically explored the molecular mechanism and microbial regulatory role of traditional Chinese medicine propolis in resisting *C. parvum* infection, and for the first time confirmed that propolis can exert anti-infection effects through multiple mechanisms. At the protein level, propolis significantly upregulates the expression of intestinal barrier tight junction proteins (occludin, ZO-1, claudin-1) and immunomodulatory factors (IL-18, NLRP6, caspase-1), enhancing the host’s defense function. Propolis’s active ingredients bind to *C. parvum* adhesion protein GP40/15 with high affinity, according to molecular docking adhesion protein GP40/15, and may block pathogen infection by binding to the adhesion protein. Further analysis of the intestinal microbiota revealed that propolis can reshape the disordered microecosystem, specifically regulate the abundance of key microbiota such as Bacteroidota and *Turicibacter*, and restore the balance of the microbiota. In the future, we can systematically isolate and identify the active components present in ABP and AWBP and determine the key compounds responsible for their insecticidal, anti-inflammatory, and microbiota-regulating effects. Furthermore, the critical role of the Nlrp6 pathway in mediating the insecticidal effects of propolis extract can be validated using gene knockout animal models, such as Nlrp6^−/−^ mice.

## MATERIALS AND METHODS

### Alcohol and aqueous extracts of propolis

The alcohol extract of propolis was obtained from Yuan Ye Biotechnology Co., Ltd., while the water extract was sourced from Pu Xitang Biotechnology Co., Ltd. Fresh propolis, a sticky substance collected by bees from plant resins and spores, undergoes alcohol extraction during processing. Following freezing and crushing, multi-step extraction is carried out using food-grade solvents such as ethanol or propylene glycol to effectively remove waxes and impurities. Subsequently, the solvent is evaporated through low-temperature vacuum concentration, ensuring the preservation of active compounds, including flavonoids and phenolic acid derivatives. The resulting extract is then converted into a uniform powder via spray drying or freeze-drying techniques. Throughout the process, temperature is strictly controlled (typically ≤60℃) to prevent thermal degradation of sensitive components.

In the production of water-soluble propolis extract, the selected raw materials are first frozen and crushed. The lipid-based matrix of propolis is then disrupted through hot water extraction (60–80℃), combined with enzymatic hydrolysis using enzymes such as cellulase or pectinase, thereby enhancing the solubility of water-extractable constituents. Insoluble impurities are subsequently removed via centrifugation or membrane filtration. Finally, the purified extract is concentrated under vacuum or dried via spray drying to obtain the water-soluble propolis extract.

### *In vitro* cytotoxicity detection and immunofluorescence of drugs

A water bath set at 37°C was used to quickly thaw the cryopreserved HCT-8 cells. The medium used to cultivate the cells was RPMI-1640 (Solaibao Technology Co., Ltd.), added to 10% fetal bovine serum (Gibco, USA) and 1% penicillin-streptomycin solution (Solaibao Technology Co., Ltd.). Cells were expanded to the 2nd–3rd passage for subsequent experimental use. The cytotoxicity of the drugs was assessed using the WST-1 assay.

Drug concentrations were set in a gradient ranging from 0 to 5,000 µM, with six replicates per concentration. Cells in the logarithmic growth phase were seeded into 96-well plates and allowed to adhere for 24 hours. Subsequently, different concentrations of BP and WBP solutions were added, followed by incubation for an additional 24 hours. After adding 10% of the WST-1 reagent to each well, the plates were left to incubate for 1–4 hours at 37°C in the dark. At 450 nm (reference wavelength: 630 nm), absorbance was determined with an ELISA reader. Drug-induced cytotoxicity was evaluated by comparing the optical density (OD) values of the treated groups with those of the untreated control group (three replicates).

Following cytotoxicity assessment, the inhibitory effect of the drugs on *C. parvum* proliferation was analyzed. Briefly, cells in the logarithmic growth phase were seeded into 96-well plates and allowed to adhere for 24 hours. Each well was then inoculated with approximately 4 × 10⁴ oocysts and exposed to varying concentrations of BP and WBP. Six replicates were included for each drug concentration. The plates were incubated at 37°C under 5% CO₂ for 48 hours. Afterward, the supernatant was removed, and the cells were fixed with a methanol-acetic acid solution (9:1 ratio) for 5 minutes. The fixed samples were washed twice with a permeabilization buffer containing 0.1% Triton X-100, 0.35 M NaCl, and 0.13 M Tris-base (pH 7.6). Nonspecific binding was blocked by incubating the samples with 5% normal goat serum for 45 minutes. Following blocking, the anti-*C*. *parvum* antibody (SporoGlo) was added, and the samples were incubated overnight at 4°C. On the following day, the samples were washed twice with PBS and once with deionized water. An inverted fluorescence microscope was used to take fluorescence images, and ImageJ software was used to measure the intensity of the fluorescence (version 1.37v).

### Animal experiment

This study was approved by the Animal Ethics Committee of Nanjing Agricultural University and employed an immunosuppressed mouse model to evaluate the therapeutic efficacy of propolis alcohol extract and aqueous extract against *C. parvum* infection. The study involved the selection and random assignment of 40 4-week-old male ICR mice from Qinglongshan Laboratory Animal Center to four experimental groups (*n* = 10): propolis alcohol extract treatment group (BPZ, 100 mg/kg), propolis aqueous extract treatment group (WBPZ, 100 mg/kg), infection control group (MZ), and control group (CZ). An immunosuppressive state was induced 3 days prior to infection by administering dexamethasone (1 mg/L) in the drinking water continuously. On the day of infection (Day 0), each mouse was orally inoculated with 200,000 *C*. *parvum* oocysts via gavage and subsequently treated with the respective propolis extracts for eight consecutive days. Daily clinical measurements, such as body weight and diarrhea, were made during the trial, and fecal samples were taken to extract DNA later on. The mice underwent anesthesia, cervical dislocation, euthanasia, and necropsy on the ninth day after infection. Blood samples were collected, and tissue samples from the jejunum and ileum were dissected. Additionally, rectal contents were harvested for further analysis.

### Quantitative analysis of oocysts in mouse feces

The feces of mice were collected every day, and the fecal DNA of mice was extracted using the fecal DNA extraction kit (Solaibao Technology Co., Ltd.). Real-time fluorescence quantitative PCR analysis was performed using specific primers targeting the *C. parvum* 18S rRNA gene (forward 5′-CTGCGAATGGCTCATTATACA-3′, reverse 5′-AGGCCAATACCCTACCGCTT-3′). The reaction system includes SYBR Green Master Mix (Vazyme Biotechnology Co., LTD.), 1 µL each of the upstream and downstream primers, and 5 µL of fecal DNA. For each sample, three technical replicates were set up. At the same time, a standard curve (10^2^–10^8^ gradient dilutions after purification of the standard *C. parvum* PCR product) was established for absolute quantification to calculate the number of *C. parvum* oocysts in feces.

### Detection of serum factors

Blood was drawn from the mice’s orbital venous plexus. It was centrifuged for 15 minutes at 4°C and 1,500 rpm to extract the serum after an hour at room temperature. Inflammatory factors and oxidative stress indicators in serum were detected using commercial ELISA kits. The concentrations of IL-6, IL-1β, TNF-α, IL-10, and IL-18 were determined using the kits provided by Solaibao Technology Co., Ltd. Meanwhile, the reagent kits from Nanjing Jiancheng Institute of Bioengineering were used to detect oxidative stress-related indicators such as SOD activity, MDA content, T-AOC, and GSH-Px activity. Every detection procedure was performed precisely in compliance with each kit’s instructions. To guarantee the accuracy of the data, three duplicate wells were set up for every sample.

### Intestinal pathological section

The number of oocysts was evaluated by RT-qPCR. The jejunum and ileum were determined as the observation sites, and the observation period was on the 9th day after infection. Mice with ICR had their small intestines removed and preserved for 48 hours at 4°C using 4% paraformaldehyde fixative. Subsequently, paraffin embedding treatment and HE staining were carried out. All stained sections were observed for histopathology using the GX41 optical microscope produced by Olympus Corporation of Japan. The primary pathological indicators that were assessed were the depth of crypts, the height of villi, the degree of inflammatory cell infiltration, and the structural integrity of the intestinal mucosa.

### Quantitative analysis of intestinal tissue genes by RT-qPCR

Approximately 100 mg of mouse jejunum and ileum tissues was collected. Total RNA was extracted by homogenizing the tissues with RNA extraction reagent (Solaibao Biotechnology Co., Ltd.), followed by purification using an RNA extraction kit (Aikerui Biotechnology). cDNA was synthesized via reverse transcription (Yisheng Biotechnology Co., Ltd.).

For RT-qPCR, cDNA was amplified using SYBR Premix Ex Taq II (Yisheng Biotechnology Co., Ltd.) with three technical replicates per sample. The β-actin gene served as the internal control, and target gene expression levels were quantified using the 2^−ΔΔCt^ method. All procedures were performed under RNase-free conditions.

### Intestinal Western blot protein quantification

Approximately 100 mg of mouse intestinal tissue was placed in pre-chilled RIPA lysis buffer (containing 1% PMSF protease inhibitor) and thoroughly homogenized using a tissue homogenizer on ice. The homogenate was then incubated at 4°C for 30 minutes to ensure complete protein extraction, followed by centrifugation at 12,000 rpm for 15 minutes at 4°C. The total protein extract was obtained from the supernatant. The protein concentration of each sample was computed using a standard curve, and protein concentration was assessed using a BCA protein assay kit (Beyotime Biotechnology) in accordance with the manufacturer’s instructions. All samples were adjusted to a uniform concentration (2 µg/µL) using 5× SDS loading buffer and denatured by boiling at 100°C for 5 minutes.

For Western blot analysis, 4–12% SDS-PAGE gel electrophoresis was used to separate 30 µg of protein per sample, which was then wet transferred to a polyvinylidene fluoride membrane. After blocking the membrane for 2 hours at room temperature with 5% non-fat milk, primary antibodies (ZO-1, occludin, NLRP6, IL-18, caspase-1, and β-actin, 1:2,000 dilution, ABclonal Biotechnology Co.) were incubated at 4°C for the entire night. The membrane was incubated with HRP-conjugated secondary antibodies (1:5,000 dilution) for 1 hour at room temperature following three TBST washes. β-Actin was used as the internal reference for normalization when protein bands were visualized using an ECL chemiluminescent substrate, and band intensity was measured using ImageJ software.

### Fecal bioinformatics sequencing analysis

Microbial genomic DNA was extracted using the QIAamp Fast DNA Stool Mini Kit (Qiagen). After determining the DNA concentration using Nanodrop, the hypervariable regions of the 16S rRNA gene V3-V4 were amplified with the universal primers 338F (5′-ACTCCTACGGGAGGCAGCAG-3′) and 806R (5′-GGACTACHVGGGTWTCTAAT-3′). The PCR system consists of 2× Taq Master Mix 15 µL, primers 1 µL each, and template DNA 10-20 ng. After the amplification products were verified by 2% agarose gel electrophoresis, the target bands were purified and recovered. Double-ended sequencing (2 × 250 bp) was performed on the Illumina NovaSeq platform. The original data, after quality control by Trimmomatic, were analyzed using the QIIME2 process for OTU clustering, species annotation, and diversity analysis. Bioinformatics analysis was conducted on the purified 16S rRNA gene amplicon sequencing data. First, the original data were subjected to quality control processing using QIIME2. The low-quality sequences (with a quality value of *Q* < 20) were removed, primer sequences were intercepted and denoised through the DADA2 algorithm to obtain high-quality non-repetitive sequences (ASVs). Subsequently, the Silva 132 database was used to annotate the representative sequences by species (with a similarity threshold set at 97%), and the microbial community composition at each taxonomic level (phylum, class, order, family, and genus) was statistically analyzed. The α-diversity index (including Shannon index, Simpson index, Chao1 index, and observed species) was calculated based on the abundance table to assess the microbial diversity within the sample. And the differences in community structure between groups were visualized through β-diversity analysis (Bray-Curtis distance, UniFrac distance) combined with principal coordinate analysis or NMDS analysis. Further LEfSe analysis (LDA > 2.0) was used to screen for landmark species with significant differences between groups, and PICRUSt2 was used to predict the functional potential of the microbial community.

### Molecular docking

Molecular docking analysis of flavonoids (acacetin, apigenin, cinnamic acid, ferulic acid, hesperidin, luteolin, quercetin, resveratrol, rutin) in propolis with *C. parvum* GP40/15 adhesion protein was conducted using AutoDock Vina to evaluate their binding characteristics.

### Data analysis

The statistical analysis of experimental data was conducted with GraphPad Prism 10.1. Measurement data were presented as mean ± standard error (mean ± SEM) using two software programs. For group comparisons, a one-way ANOVA was employed. Statistical significance was defined as a *P* value < 0.05. The independent sample *t*-test was employed to compare the two groups. The GraphPad Prism software is designed to ensure that the chart presentation is standardized and accurate.

## Data Availability

The NCBI Sequence Read Archive database contains all animal raw sequence data under accession number PRJNA1297616.
